# Novel *GNAO1* variant in α-helical domain reveals alternative mechanism of disease

**DOI:** 10.1016/j.gendis.2025.101714

**Published:** 2025-11-21

**Authors:** William Grant Ludlam, Jana Domínguez-Carral, Angeles Schteinschnaider, Kirill A. Martemyanov, Juan Darío Ortigoza-Escobar

**Affiliations:** a Department of Neuroscience, The Herbert Wertheim UF Scripps Institute for Biomedical Innovation & Technology, University of Florida, Jupiter, FL 33458, USA; b Epilepsy Unit, Department of Child Neurology, Coordinating member of the ERN EpiCARE, Hospital Sant Joan de Déu, Universitat de Barcelona, Barcelona 08950, Spain; c Department of Pediatric Neurology, FLENI Institute, Buenos Aires, C1428 AQK, Argentina; d Movement Disorders Unit, Department of Child Neurology, Institut de Recerca Sant Joan de Déu, Barcelona 08950, Spain; e U-703 Center for Biomedical Research on Rare Diseases (CIBER-ER), Instituto de Salud Carlos III, Barcelona 08950, Spain; f European Reference Network for Rare Neurological Diseases (ERN-RND), Barcelona 08950, Spain

G protein subunit alpha O1 (*GNAO1*)-related disorders (*GNAO1*-RD) are a group of ultra-rare neurological conditions characterized by a wide spectrum of clinical features, including movement disorders, developmental delay or intellectual disability, epilepsy, and feeding difficulties. The severity of these conditions can range from mild to severe,^1^^,^^2^ with life-threatening episodes of dyskinetic crisis being a hallmark of the disorder in many cases.^3^ The Gαo protein, encoded by the *GNAO1* gene, is a crucial component of G protein-coupled receptor (GPCR) signaling. It mediates interactions between receptors and intracellular effectors, playing an essential role in neuronal communication and regulation. Structurally, Gαo consists of a Ras-like domain (RHD) and an α-helical domain (AHD), which coordinate nucleotide binding and signaling, along with an N-terminal α-helix (αN). While mutations in the RHD are well-studied due to their role in enzymatic activity and interactions, the functional impact of mutations in the AHD, such as the novel N76K variant of *GNAO1*, is less understood. Here, we report and analyze the N76K variant, focusing on its effects on Gαo activity and signaling pathways.

The patient is an 8-year-old girl with developmental delay, epilepsy, and movement disorders, including a history of dystonic status. She was born at term to healthy, non-consanguineous Argentinian parents, with no significant family history of neurological disorders apart from distant relatives with neurodevelopmental delay. Hypotonia and motor delay were noted at 6 months. At 1 year, she experienced three febrile seizures but remained seizure-free after two years of phenytoin treatment. Generalized motor seizures recurred at 5 years, but no antiseizure medication was initiated; no further seizures have occurred. At age 6, she developed severe choreoathetosis and dystonia, progressing to refractory status dystonicus requiring ICU care, tracheostomy, and gastrostomy. She is currently on trihexyphenidyl and tetrabenazine. A *GNAO1* severity score of 9.75 reflects her severe phenotype.^1^ Brain magnetic resonance imaging showed nonspecific periventricular gliosis, and genetic testing revealed a *de novo* likely pathogenic *GNAO1* variant (c.228A>C/p.N76K), classified as likely pathogenic according to the guidelines of the American College of Medical Genetics and Genomics (PP3, PM1, and PM2). This variant localizes to the αA helix of the α-helical domain, a region critical for stabilizing interactions within the Gαo protein.

The study was conducted in accordance with ethical standards and was approved by the Ethics Committee of Institut de Recerca Sant Joan de Deu, under protocol number PIC-150-23. To investigate the molecular mechanism underlying the pathology of the N76K variant, we examined its location and function within the Gαo protein structure (Fig. 1A). The N76K substitution is found in the αA helix of the α-helical domain, a region conserved across Gα subtypes despite low sequence homology (Fig. 1B). We first evaluated whether the variant affected protein stability or expression levels. Immunoblotting in HEK cells revealed a trend toward reduced Gαo expression in the N76K variant compared with wild-type (WT), although this difference was not statistically significant (Fig. 1C). While these results do not support a major impact on protein stability, a subtle effect on expression levels cannot be excluded.

**Figure 1:**
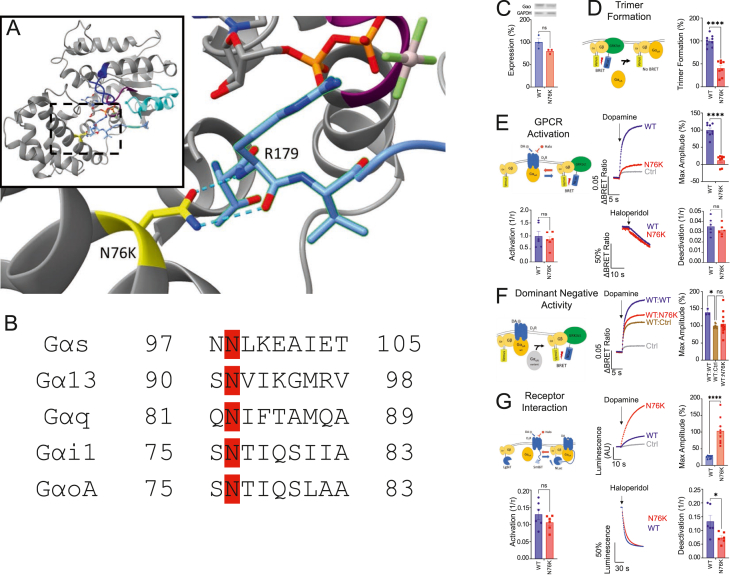
Characterization of GNAO1 N76K variant and its effects on Gαo function. **(A)** Location of N76 on Gαo (PDB: 3C7K). Regions are indicated as follows: purple for P-loop; cornflower blue for Switch I; orange for GDP phosphates; yellow for N76. **(B)** Alignment of representative members of different Gα subtype families showing the conserved asparagine in the αA helix. **(C)** Expression level of *GNAO1* N76K variant. HEK293FT cells were transfected with WT or N76K Gαo or pcDNA control, lysed, and then immunoblotted with α-Gαo antibody (*n* = 3 independent experiments). **(D)** The effect of *GNAO1* N76K variant on basal BRET. The 0% heterotrimer formation was defined as the amount of basal BRET without Gαo expression. The degree of heterotrimer formation was calculated by subtracting the basal BRET value of WT or N76K Gαo expressing cells from the Gαo-free control. The results were normalized to the WT Gαo control (*n* = 9 technical replicates across three independent experiments). **(E)** Schematic of the G protein-coupled receptor (GPCR) signaling assay. GPCR activation allows Gαo to release Venus-tagged Gβγ, which then interacts with NanoLuc-tagged GRK, causing a shift in the BRET ratio. Representative traces of dopamine BRET responses are shown. The maximum amplitude of the change in BRET signal was measured in comparison to the average baseline BRET value (*n* = 9 technical replicates across three independent experiments). Activation rate constants were calculated for traces (*n* = 6 technical replicates across two independent experiments). Representative traces of haloperidol deactivation and calculated rate constants are shown (*n* = 6 technical replicates across two independent experiments). **(F)** The effect of the *GNAO1* N76K variant on the WT Gαo dopamine-induced BRET signal. The 100% activity was defined as the maximum amplitude of the condition expressing pcDNA alongside WT Gαo (WT:Ctrl). Representative traces are shown (*n* = 3–12 technical replicates across 1–4 independent experiments). **(G)** The effect of the *GNAO1* N76K variant on dopamine-induced receptor interaction with G protein heterotrimer. Representative luminescence traces are shown. Runs were normalized by taking the amount of luminescence detected after stimulation of the Gαo-free control with dopamine as 0% interaction and the amount of luminescence detected with WT Gαo as 100% interaction. A luminescent readout is given after the SmBiT tag on D2R interacts with the LgBiT tag on Gβ and reconstitutes NanoLuc (*n* = 9 technical replicates across three independent experiments). Activation rate constants were calculated for traces (*n* = 6 technical replicates across two independent experiments). Representative traces of haloperidol deactivation and calculated rate constants are shown (*n* = 6 technical replicates across two independent experiments).

Using a bioluminescence resonance energy transfer (BRET) assay, we assessed the ability of Gαo to associate with G protein βγ (Gβγ) subunits and release them upon GPCR activation. The N76K variant displayed increased baseline BRET values, suggesting impaired association with Gβγ (Fig. 1D). However, given considerable variability inherent to baseline measurements, caution should be taken when interpreting the magnitude of the effect. To further explore this, we used the dopamine 2 receptor (D2R) as a model GPCR. Following dopamine stimulation, the N76K variant exhibited a reduced maximal response compared with WT, although activation and deactivation rates were similar (Fig. 1E). This indicates that the variant does not disrupt signaling dynamics but reduces overall signaling capacity.

We also tested whether N76K exerted dominant-negative effects by co-expressing it with WT Gαo (Fig. 1F). The presence of the variant did not diminish the WT dopamine response, indicating normal WT function alongside N76K. Finally, we examined Gβγ recruitment to D2R using NanoBiT technology (Fig. 1G). The N76K variant caused a fourfold increase in receptor-Gβγ interaction, suggesting altered binding dynamics. While the activation rate was unchanged, a slight decrease in deactivation rate was observed following antagonist treatment. These findings demonstrate that the N76K variant disrupts Gαo signaling by altering Gβγ interactions and receptor coupling, rather than through dominant-negative mechanisms or protein instability. Detailed information regarding cell culture and transfection, BRET assay, NanoBiT assay, quantification and statistical analysis, and Gα sequence alignment can be found in the supplementary materials and methods.

The N76K mutation replaces asparagine with lysine within the α-helical domain. The asparagine residue forms critical hydrogen bonds with the catalytic arginine (R179) in the RHD, stabilizing the nucleotide-bound state. Disruption of this bond likely destabilizes the interaction between the domains, altering nucleotide retention and signaling dynamics. Structural studies suggest that N76K causes rapid nucleotide exchange, leading to increased basal activity but reduced activation efficiency in response to receptor stimulation.

Functional assays revealed that the N76K variant decreased the affinity for Gβγ subunits in the basal state and reduced the efficiency of Gβγ release upon GPCR activation, in line with previous findings suggesting impaired Gαo cycling dynamics.^4^ Moreover, the increased interaction between Gβγ and the receptor observed in our assay supports earlier reports of altered G-protein coupling due to N76K and may reflect a compensatory mechanism or abnormal stabilization of the GPCR-Gβγ complex.^4^ Although these changes disrupt normal signaling, the absence of a dominant-negative effect on WT Gαo activity indicates that the N76K retains partial functionality, as previously described.

The distinct mechanism of N76K contrasts with other variants, such as K46E, located in the GDP/GTP-binding region.^5^ K46E prevents effective nucleotide binding, leading to stable but inactive complexes, while N76K causes dynamic instability between active and inactive states. This highlights the diverse pathological mechanisms within *GNAO1*-RD, emphasizing the need for variant-specific therapeutic approaches.

Clinically, the N76K variant is associated with severe *GNAO1*-RD, including developmental delay, epilepsy, and movement disorders. The phenotype resembles RHD-related variants more than αN-helix variants like L13P and L23P, which are linked to Parkinsonism. Establishing these phenotype–genotype correlations is critical for advancing diagnosis and treatment.

This study expands the spectrum of pathogenic *GNAO1* variants and underscores the essential role of the α-helical domain in Gαo function and signaling. By identifying the unique signaling profile of the N76K variant, we provide novel insights into *GNAO1*-RD pathophysiology and inform future therapeutic strategies tailored to specific variant mechanisms.

## CRediT authorship contribution statement

**William Grant Ludlam:** Writing – review & editing, Writing – original draft, Validation, Methodology, Formal analysis. **Jana Domínguez-Carral:** Writing – review & editing, Data curation. **Angeles Schteinschnaider:** Data curation. **Kirill A. Martemyanov:** Writing – review & editing, Validation, Supervision, Methodology, Formal analysis, Data curation, Conceptualization. **Juan Darío Ortigoza-Escobar:** Writing – original draft, Methodology, Data curation, Conceptualization.

## Data availability

The data that support the findings of this study are available from the corresponding authors upon reasonable request.

## Funding

This work was funded by the 10.13039/100000002US National Institutes of Health grants DA036596 and DA048036 to K.A.M.

## Conflict of interests

The authors declared no conflict of interests.
