# Supplementary material for: Novel GNAO1 variant in α-helical domain reveals alternative mechanism of disease
Source: Genes Dis. 2025 Nov 21;13(2):101714. doi: 10.1016/j.gendis.2025.101714 (PMC12682024; doi:10.1016/j.gendis.2025.101714)
Supplement: Multimedia component 1 [file mmc1.docx]

# Material and Methods

**Cell culture and transfection**

HEK293FT cells were grown in DMEM supplemented with 10% FBS5, non-essential amino acids, and 1 mM sodium pyruvate at 37°C in a humidified incubator containing 5% CO_2_. At the time of transfection, cells were supplemented with 0.1% Matrigel (Corning).

For bioluminescence resonance transfer (BRET) assays, cells were seeded in 96-well flatbottomed white microplates (Greiner Bio-One) at a density of 5 × 10^4^ cells/well. At the same time the cells were plated, they were transfected with expression constructs (total 0.09 μg/well), PLUS reagent (0.1 μL/well), and Lipofectamine LTX (0.5 μL/well). The expression constructs transfected were as follows (number in parentheses indicates the relative amount of DNA, where 1 = 0.015 μg): Flag-D2R (1), GαoA (2), Venus 156–239-Gβ1 (1), Venus 1–155Gγ2 (1), and masGRK3ct-Nluc-HA (1).

For NanoBiT assays, cells were seeded into 6-cm dishes at a density of 4 × 10^6^ cells/dish. After 4 hr., expression constructs (total 2.1 μg/dish) were transfected into the cells using PLUS reagent (7.5 μL/dish) and Lipofectamine LTX (12 μL/dish) reagents. The expression constructs transfected were as follows (number in parentheses indicates the relative amount of DNA, where 1 = 0.42 μg): D2R-mycSmBiT (1), GαoA (1), LgBiT-Gβ1 (1), and Gγ2 (1).

**BRET assay**

BRET between Venus-Gβ1γ2 and masGRK3ctNluc-HA was used to measure trimer formation, agonist-induced G protein activation, and dominant-negative activity of Gαo mutants in living cells. 16 to 24 hr. post-transfection, cells were washed once with BRET buffer (Phosphate-Buffered Saline (PBS) containing 0.5 mM MgCl_2_ and 0.1% glucose). Cells were harvested with centrifugation at 500 g for 5 min and resuspended in BRET buffer. The substrate for Nano luciferase (Nluc), furimazine (Promega), was diluted in BRET buffer 1:750 and added to the cells. BRET measurements were made every 100 ms using a microplate reader (PHERAstar FSX; BMG Labtech) equipped with two emission photomultiplier tubes. All measurements were performed at 37 °C. The BRET signal was calculated as the ratio of the light emitted by Venus-Gβ1γ2 (535 nm ± 30 nm) over the light emitted by masGRK3ct-Nluc-HA (475 nm ± 30 nm). A baseline BRET value was obtained for 10 s. 100 μM dopamine (Sigma) was added to stimulate G protein activation for 35 s. Lastly, 100 μM haloperidol was added to quench G protein activation, and measurements were taken for another 45 s. The average baseline BRET value recorded prior to dopamine stimulation was subtracted from the experimental BRET signal values. To calculate rate constants, the greatest and lowest ΔBRET values of the decay curves were normalized to 100% and 0%, respectively. The curves were then fitted with one-phase nonlinear equations in GraphPad Prism 10.0.2.

**NanoBiT assay**

Measurement of bioluminescence caused by complementation between D2R-SmBiT and LgBiT-Gβ1 was performed to examine the interaction between G proteins and the receptor. 16 to 24 h post-transfection, HEK293FT cells were washed once with BRET buffer and detached by gentle pipetting. Approximately 5-10 × 10^4^  cells/well were transferred to a 96-well flatbottomed white microplate (Greiner Bio-One). The substrate for Nano luciferase (Nluc), furimazine (Promega), was diluted in BRET buffer 1:750 and added to the cells. Luminescence measurements were made every 0.74 s using a microplate reader (PHERAstar FSX; BMG Labtech) equipped with two emission photomultiplier tubes. All measurements were performed at 37 °C. A baseline luminescence value was obtained for 5 s after addition of furimazine. 100 μM dopamine (Sigma) was added to stimulate G protein activation, and measurements were taken for 85 s. 100 μM haloperidol was then added to quench G protein activation, and measurements were taken for an additional 72.8 s. To calculate rate constants, the greatest and lowest ΔBRET values of the decay curves were normalized to 100% and 0%, respectively. The curves were then fitted with one-phase nonlinear equations in GraphPad Prism 10.0.2.

**Quantification and statistical analysis.**

For functional studies, samples with only pcDNA3.1+ transfected in place of Gαo were used as baseline measurements. The max amplitude of these measurements was subtracted from the max amplitude of all other measurements. Statistical analysis was performed using GraphPad Prism 10.0.2. All data are represented as mean ± SEM. Comparisons were computed using two-tailed unpaired Student’s t-test for pairwise comparisons and one-way ANOVA for comparisons involving more than two groups. Asterisks indicate statistical significance (* = p < 0.05, ** = p < 0.01, *** = p < 0.001, **** = p < 0.0001).

**Gα sequence alignment.**

The sequences of five representative human Gα subtypes (Gαi1, P63096; Gαs long, P63092; Gα13, Q14344; Gαq, P50148; GαoA, P0947) were aligned using the Clustal Omega (1.2.4) web tool.
